# Trapped: A Molecular-Level Look at How Herpes Disables Its Immune Destroyer

**DOI:** 10.1371/journal.pbio.0040189

**Published:** 2006-05-02

**Authors:** Mary Hoff

It's not easy coming out ahead in the virus–host battle: the mechanisms living things have for seeking and destroying invaders, as elaborate as they might be, often find themselves up against equally elaborate viral mechanisms for evading them.

Such is the case when it comes to humans versus herpes simplex virus type-1 (HSV-1), a highly contagious virus that is virtually impossible to get rid of once it becomes established. When our bodies detect HSV-1 inside us, they mass produce antibodies called immunoglobulin G (IgG). These Y-shaped defense molecules latch onto antigens on the surface of the virus and virus-infected cells with their two arms, disabling them and marking them for pickup by the immune-system equivalent of roving trash trucks. But HSV-1 has a cagey way of turning the tables. A protein pair that it stations on its surface (and on the surface of cells it has taken over) known as gE-gI can grab IgG by its tail (known formally as its Fc region), incapacitating it instead.

Elizabeth Sprague, Pamela Bjorkman, and colleagues explored this interaction by focusing their attention on CgE, a portion of gE. Using an assay that measures how well two substances stick to each other, they showed that CgE binds Fc even in the absence of gI or other gE domains. They then used a technique that compares how crystals of CgE diffract X rays of various wavelengths to determine the three-dimensional structure of CgE.

Next the researchers attempted to create a three-dimensional picture of the gE-gI/Fc complex. Using the new information about CgE's structure, low-resolution images of gE-gI/Fc crystals, and techniques that make it possible to infer complete structure from partial information, they proposed a structure for the gE-gI/Fc complex in which two CgE components of gE-gI hold Fc like two hands holding a basketball. To substantiate that surmised structure, they applied a computational approach called protein docking, using what they knew about the structure of unbound CgE and Fc to calculate what complex would be energetically most favorable. Of the five plausible models they came up with, one was remarkably similar to the structure derived through the previous process, confirming its likelihood as the correct structure.

The researchers then used the newfound structural information to further explore the gE-gI/Fc interaction. They compared CgE with other proteins and a peptide of known structure that bind with the same region of Fc. They also used the structural information to explain on a molecular level the previously known inability of an Fc mutant, another human immunoglobulin, and rodent IgG to bind with gE-gI.

One fascinating mystery the structures helped the researchers address is the fact that gE-gI binds well with Fc at neutral or slightly basic pH, but not in an acidic environment. The researchers showed that CgE-Fc binding is pH dependent, and attributed this trait to four histidine amino acids at the CgE/Fc interface that would likely alter the complex's chemistry in the presence of spare protons. They also speculated that the pH sensitivity was part of a viral strategy for attacking IgG in which gE-gI/IgG is drawn into the cell where the acidity causes the antibody to dissociate and eventually to be destroyed.

The structural studies also shed light on previous findings regarding the effects of mutations in gE on its ability to bind IgG and on cell-to-cell spread of HSV-1. Using their knowledge of CgE and gE-gI/Fc structures, the researchers were able to identify the structural and functional implications of various mutations and to pinpoint specific regions of CgE implicated in cell-to-cell spread.

Finally, the researchers used the structures to determine that gE-gI/IgG complexes likely orient upright in the cell membrane, leaving the arms of the gE-gI-bound IgG available to attach to a nearby HSV-1 antigen—a previously hypothesized state known as bipolar bridging. If bipolar bridging does indeed occur, subsequent endocytosis would then swallow up not only IgG but also any antigens involved in bridging, further hindering efforts to win the battle against this tricky viral invader.

## 

**Figure:**
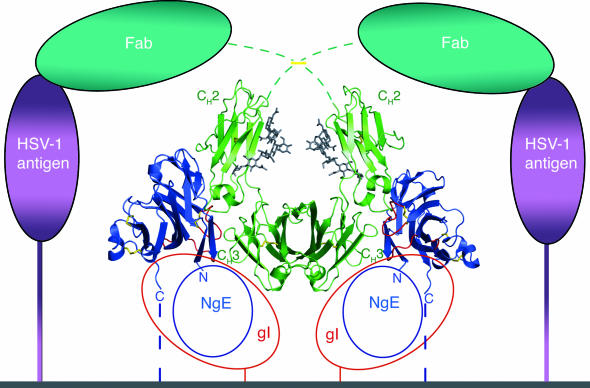
The three-dimensional structure of a gE-gI/Fc complex suggests that an anti-HSV IgG bound by gE-gI is in an upright orientation, positioning it to also bind its antigen on the surface of the same cell.

